# Upcycling of Degraded Prussian Blue into Layered Materials for Sodium-Ion Battery

**DOI:** 10.34133/research.0643

**Published:** 2025-03-21

**Authors:** Weng-Lam Wong, Jiahui Xu, Yun Zhao, Yadong Wang, Hao Du, Junhao Zhang, Yuqiong Kang, Yuqing Chen, Feiyu Kang, Baohua Li

**Affiliations:** ^1^ Institute of Materials Research, Tsinghua Shenzhen International Graduate School, Tsinghua University, Shenzhen 518055, China.; ^2^ Key Laboratory of Pollution Exposure and Health Intervention of Zhejiang Province, Interdisciplinary Research Academy, Zhejiang Shuren University, Hangzhou 310021, China.

## Abstract

Prussian blue and Prussian blue analogs are widely used in sodium-ion batteries (SIBs). In this study, we upcycle the degraded Prussian blue directly into layered materials for SIBs through thermal treatment. Prussian blue thermally decomposes to form metal oxides, which then recrystallize into layered metal oxides with metal–nitrogen bond on their surface under suitable temperature conditions. This transformation method is similar to solid-state synthesis, allowing for the pre-addition of necessary components before material conversion to optimize the composition and integrity of the target materials. Based on in situ x-ray diffraction observations of the crystal structure changes of Prussian blue at different temperatures, we demonstrate 1,000 °C as the optimal temperature for converting to layered materials. These materials exhibit an initial discharge capacity of 122.3 mAh g^−1^ and good rate and cycling stability. We hope that this research will promote the sustainable development of the SIB industry.

## Introduction

The development of sustainable energy requires batteries to address their intermittent nature. Although lithium-ion batteries (LIBs) have rapidly advanced over the past 3 decades, their widespread use has been limited due to resource constraints and cost issues [1,2]. However, some applications do not necessarily require superior performance provided by LIBs [3]. As a result, a variety of alternative power sources for LIBs have emerged over the past decade, with sodium-ion batteries (SIBs) successfully commercialized in 2023. They are expected to have extensive applications in low-speed electric vehicles and energy storage systems [4].

However, SIBs are very similar to LIBs, with limited lifespans, comparable material systems, and nearly identical environmental impacts. This implies that, after approximately 10 years of use, spent SIBs must be recycled [5]. While there is still some time before SIB recycling becomes a pressing issue, the lessons learned from the recycling challenges associated with polyolefins and LIBs highlight the need for simultaneous consideration of battery development and recycling [6–10]. From a sustainable development perspective, the mindset of prioritizing development over environmental management should be abandoned [11].

Current battery recycling methods include pyro-metallurgical (Pyro), hydro-metallurgical (Hydro), direct regeneration, and upcycling methods [12]. Pyro and Hydro, as traditional metallurgical approaches, have been commercialized on a large scale and offer compatibility for recycling various battery types and materials [13,14]. However, due to the low content of precious metal elements in SIBs, traditional recycling methods often prove economically unviable. Direct regeneration and upcycling focus on the direct utilization of materials, eliminating the need for dissolution and resynthesis processes. They present clear advantages in terms of economy, environmental impact, and energy and chemical consumption [15,16]. The key difference is that direct regeneration involves the restoration of degraded materials, while upcycling transforms existing materials to meet the demands of future battery technologies [16]. They are complementary rather than competing technological pathways and can collaboratively promote material advancement and reuse. Given that SIBs are still in the early stages of large-scale application, future battery materials may be optimized or directly replaced as technology evolves [17]. Therefore, research into the transformation of existing SIBs materials is crucial for the development of future recycling technologies [5].

Prussian blue and its analogs (PB and PBAs), represented as Na_2_M_1*x*_M_2*y*_M_3*z*_[M(CN)_6_], where M stands for different transition metals, have become one of the mainstream cathode materials for SIBs. This is due to their advantages such as high theoretical capacity, an open 3-dimensional (3D) framework structure, and ease of synthesis, showing great potential for commercial applications. However, when PB materials are exposed to air for a long time, they are affected by moisture and oxygen in the air. This leads to the generation of adsorbed water, causing volume expansion of the materials and the hydrolysis of Fe(CN)_6_^4−^ groups, which damage the electrochemical performance of the materials. Here, we convert the degraded PB (D-PB) into layered cathode materials for SIBs through thermal treatment [18]. The D-PB can decompose under high-temperature conditions, and a uniform layered material is formed through solid-state crystallization. By appropriately adjusting the temperature and sodium content, an ideal layered cathode material can be obtained.

## Results and Discussion

### Failure mechanism of PB

PB exhibits a unique cubic structure, which has structural degradation upon prolonged exposure to moisture and oxygen in the air [19]. This degradation impedes the intercalation and deintercalation of sodium ions, leading to a capacity reduction of the material. We first elucidate the failure mechanism of PB because it may be important for its transformation. As shown in Fig. S1A and B, the x-ray diffraction (XRD) patterns of both PB and D-PB exhibit similar face-centered cubic (fcc) structures with the space group Fm$\bar{3}$m, featuring diffraction peaks of (200), (400), (220), and (420) [20]. Notably, the (200) and (400) peaks of the D-PB shift to lower angles compared to those of the PB, which is likely due to volumetric expansion caused by water adsorption. This expansion leads to an increased interlayer distance and lattice parameter.

In the Fourier transform infrared spectroscopy (FTIR) of both samples (Fig. S1C), the absorption peaks of D-PB and PB are found to be nearly identical, dominated by C≡N, O-H, Fe-CN, and Fe-O peaks. Furthermore, the x-ray photoelectron spectroscopy (XPS) results in Fig. S1D to F reveal that the valence states of Ni and Mn in D-PB and PB are similar, while Fe undergoes oxidation, with part of the iron being oxidized from a divalent to a trivalent state because of the decomposition of Fe(CN)_6_^4−^. This indicates that the failure process of PB in air primarily involves a Fe change.

Scanning electron microscopy (SEM) analyses of PB and D-PB reveal that water adsorption in D-PB disrupts the integrity of the cubic structure without causing a marked structural transformation (Fig. S1G). The energy-dispersive spectroscopy (EDS) results of D-PB indicate that the distributions of C, O, Na, Ni, Fe, and Mn elements are relatively uniform, confirming that water and air exposure do not significantly affect the elemental distribution of the material (Fig. S1H). These characterizations demonstrate that prolonged exposure of PB to air leads to the degradation of the cubic structure.

### D-PB transformation

**Fig. 1.**
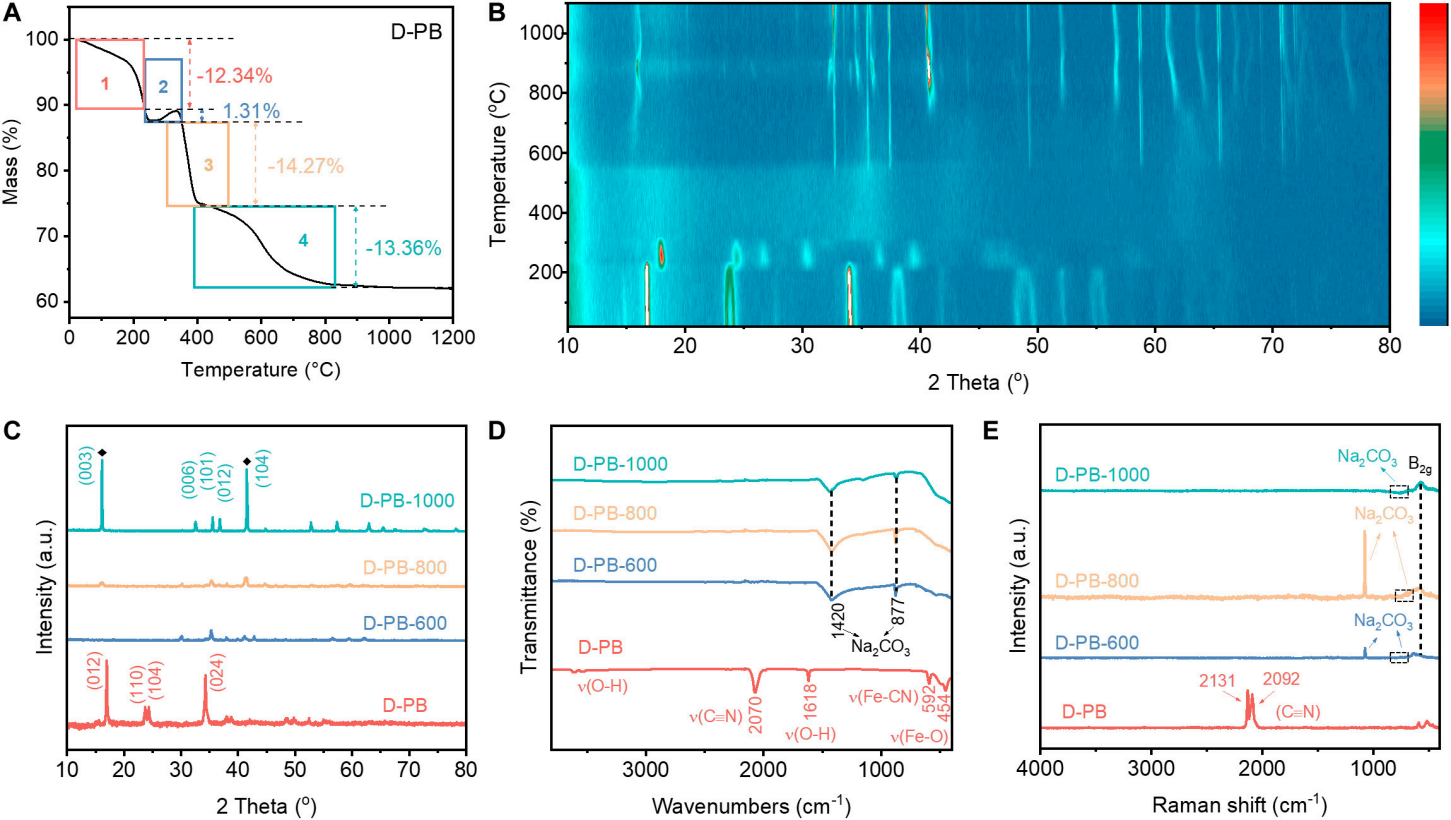
D-PB transformation. (A) TGA curves. (B) In situ high-temperature XRD spectra of D-PB. (C) XRD spectra of D-PB, D-PB-600, D-PB-800, and D-PB-1000. (D) FTIR spectra of D-PB, D-PB-600, D-PB-800, and D-PB-1000. (E) Raman spectra of D-PB, D-PB-600, D-PB-800, and D-PB-1000.

The transformation of D-PB into other materials necessitates the removal of its cyanide (CN) groups. Fortunately, the thermal instability and volatility of the CN groups make thermal treatment an effective strategy for their removal, thereby facilitating the transformation of D-PB. To determine an appropriate thermal transformation temperature for the D-PB, thermogravimetric analysis (TGA) and in situ XRD tests are conducted to monitor the mass and crystal phase change, respectively. As shown in Fig. 1A, the TGA curve of the D-PB under an air atmosphere reveals 4 distinct inflection points corresponding to mass change. These inflection points divide the TGA curve into 4 stages. In the first stage from 25 to 220 °C, the decomposition of adsorbed water within the D-PB results in a mass loss of approximately 12.34%. The second stage from 220 to 320 °C involves an oxidation reaction that forms Na_2_CO_3_, leading to a mass increase of 1.31%. The third stage from 320 to 400 °C corresponds to the decomposition of the C≡N bonds, resulting in a mass loss of approximately 14.27%. In the fourth stage from 320 to 1,000 °C, the high-temperature thermal transformation of D-PB occurs, during which the release of oxygen causes further mass loss, amounting to about 13.36%. At 1,000 °C, the thermal decomposition of D-PB reaches a stable stage, leaving a residual mass of 58.72%.

To investigate the phase changes during the thermal treatment of the D-PB, in situ XRD characterization is performed over the temperature range of 25 to 1,000 °C [21]. As shown in Fig. 1B, the D-PB can still be divided into 4 temperature stages (25 to 220 °C, 220 to 320 °C, 320 to 520 °C, and 520 to 1,000 °C). In the first stage from 25 to 220 °C, the characteristic peaks of the D-PB show no significant changes, indicating that the decomposition of adsorbed water does not affect the phase structure. In the second stage from 220 to 320 °C, the phase structure of the D-PB undergoes obvious changes. All adsorbed water is removed, leading to the collapse of the material structure, with the overall peak positions shifting to high angles.

In the third stage from 320 to 520 °C, the characteristic peaks of the D-PB disappear, suggesting that the PB structure is destroyed. In the fourth stage (520 to 1,000 °C), the diffraction peaks reappear near 600 °C, indicating the formation of a new structure. The layered structure’s characteristic peaks are obvious between 800 and 1,000 °C. These results demonstrate that a simple thermal treatment process (520 to 1,000 °C) can convert the D-PB material into a layered material. Therefore, in this study, the temperatures of 600 °C (the initial temperature at which the characteristic peaks reappear after disappearing), 800 °C (the initial temperature at which the material clearly forms a layered structure), and 1,000 °C (the temperature at which the layered structure stabilizes) are selected for the thermal transformation of the D-PB material.

The XRD patterns of the 3 thermal-treated D-PB, D-PB-600, D-PB-800, and D-PB-1000 reveal that the 2 main diffraction peaks of the D-PB-1000 sample at 16.6° and 41.4° correspond to the (003) and (104) planes of a hexagonal crystal structure, respectively, which are the characteristic of layered materials [22]. In contrast, the diffraction peaks of the layered structure in D-PB-800 are weak, and they are absent in the D-PB-600 sample [23]. These suggest that the conversion degree of the D-PB material at these 2 temperatures is low (Fig. 1C).

We then investigate the residual of specific groups in D-PB after thermal treatment as they can partly demonstrate the completeness of D-PB transformation. Compared to the FTIR results of D-PB, D-PB-600, D-PB-800, and D-PB-1000 exhibit only 2 peaks of Na_2_CO_3_ at 1,420 and 877 cm^−1^, while the characteristic peaks of C≡N, O-H, Fe=CN, and Fe-O disappear (Fig. 1D) [24]. This corresponds to the decomposition reactions occurring in the first 2 stages as observed in Fig. 1A and B. In the Raman spectra shown in Fig. 1E, D-PB-600, D-PB-800, and D-PB-1000 also exhibit the symmetric stretching vibration (~1,077 cm^−1^) and in-plane bending vibration (~634 cm^−1^) of Na_2_CO_3_. Similar to the FTIR results, the symmetric stretching vibrations of C≡N (~2,131 and 2,092 cm^−1^) are absent in all 3 samples [21]. In addition, particle size distribution tests are conducted on the materials treated at different temperatures (Fig. S2). The D50 value of D-PB-1000 is 7.77 μm. Meanwhile, it can be observed from the distribution diagram that its particle size distribution is highly uniform.

**Fig. 2.**
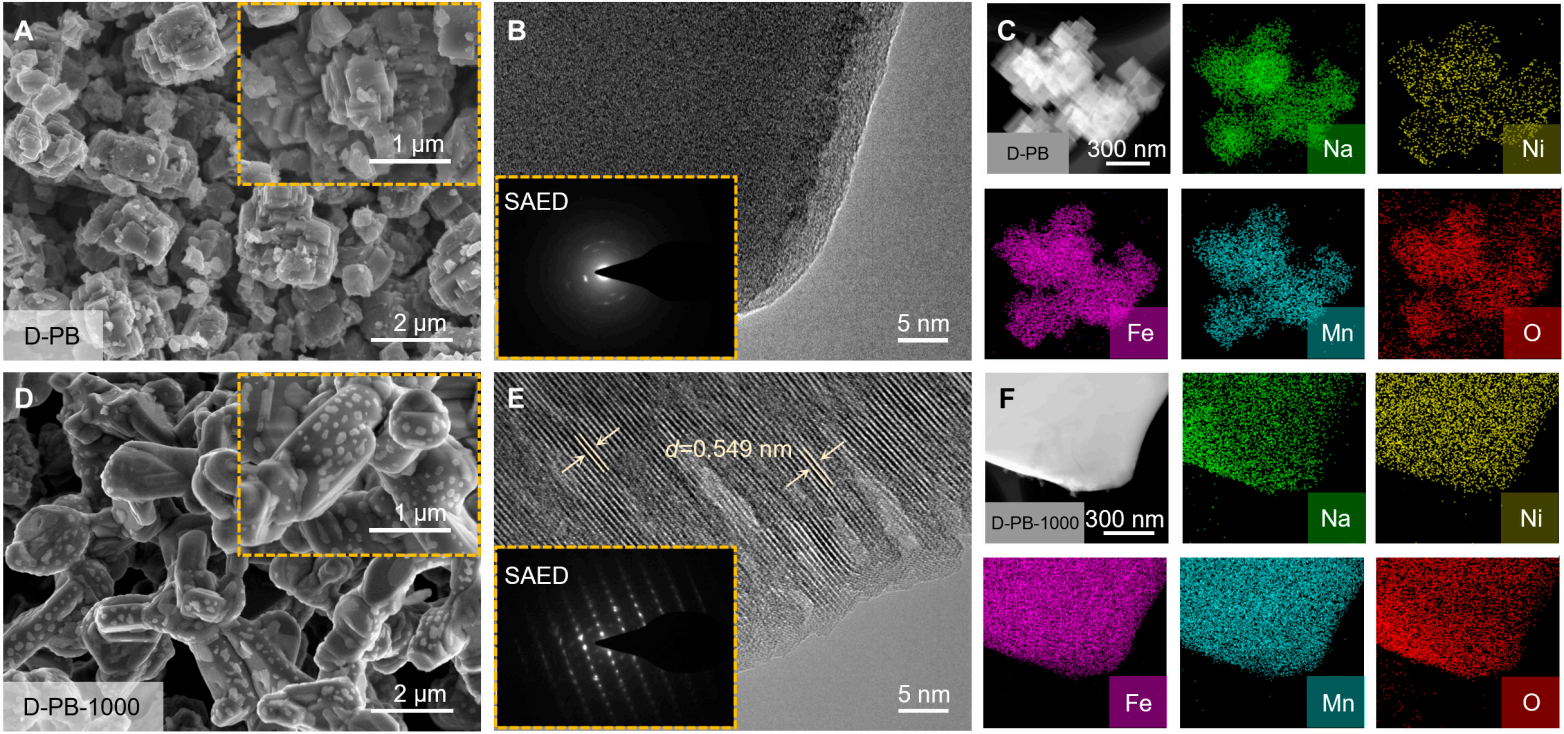
Morphology and microstructure of the transformed materials. (A) SEM images of D-PB. (B) HRTEM and SAED images of D-PB. (C) EDS mapping of D-PB. (D) SEM images of D-PB-1000. (E) HRTEM and SAED images of D-PB-1000. (F) EDS mapping of D-PB-1000.

To investigate the morphology and microstructure of the transformed materials, SEM and TEM (transmission electron microscopy) analyses are conducted (Fig. 2). The D-PB exhibits an incomplete cubic structure (Fig. 2A to C). However, it does not undergo a phase transformation and remains in a single-crystal structure. As the thermal treatment temperature increases, the D-PB material initially undergoes decomposition. This is evident from the EDS spectra, where the homogeneity of elements in the material increases in the order D-PB-600 < D-PB-800 < D-PB-1000. The EDS images reveal that the distribution of various elements in the materials is not uniform, particularly for Ni and Mn elements. At the same time, melting phenomena can be observed at the edges of the materials for D-PB-600 (Fig. S3A to C). The material exhibits multiple crystal lattice fringes with different orientations because of incomplete conversion reactions. This indicates that at 600 °C, complete transformation of the D-PB material cannot be achieved.

The SEM image of D-PB-800 exhibits a morphology similar to that of D-PB-600 (Fig. S3D). However, due to the high temperature, the material is almost completely molten and has begun to recrystallize. The TEM and high-resolution TEM (HRTEM) images reveal that the edges of the material become smoother, with 2 distinct lattice fringes. This indicates that at 800 °C, the material gradually melts but does not receive enough energy to undergo a complete structural transformation (Fig. S3E). The EDS analysis shows that Fe, Mn, and O have formed a uniform substance, while Na and Ni have not yet fully participated in the crystallization reaction (Fig. S3F).

D-PB-1000 exhibits a well-organized and uniform morphology, with particles approximately 5 μm in size (Fig. 2D). As the sodium salt has not fully undergone a solid-phase reaction with the material within 10 h, a significant amount of residual material remains on the surface. Combined with the TEM and HRTEM images (Fig. 2E), the material appears to have a relatively complete edge and a distinct lattice fringe. The measured interplanar spacing is 0.549 nm, which confirms that it belongs to the O3-phase layered material. The electron diffraction pattern confirms that it is in a single-crystal structure [25]. The EDS analysis reveals that each element is evenly distributed, demonstrating that at this temperature, D-PB can be converted into a layered material (Fig. 2F).

**Fig. 3.**
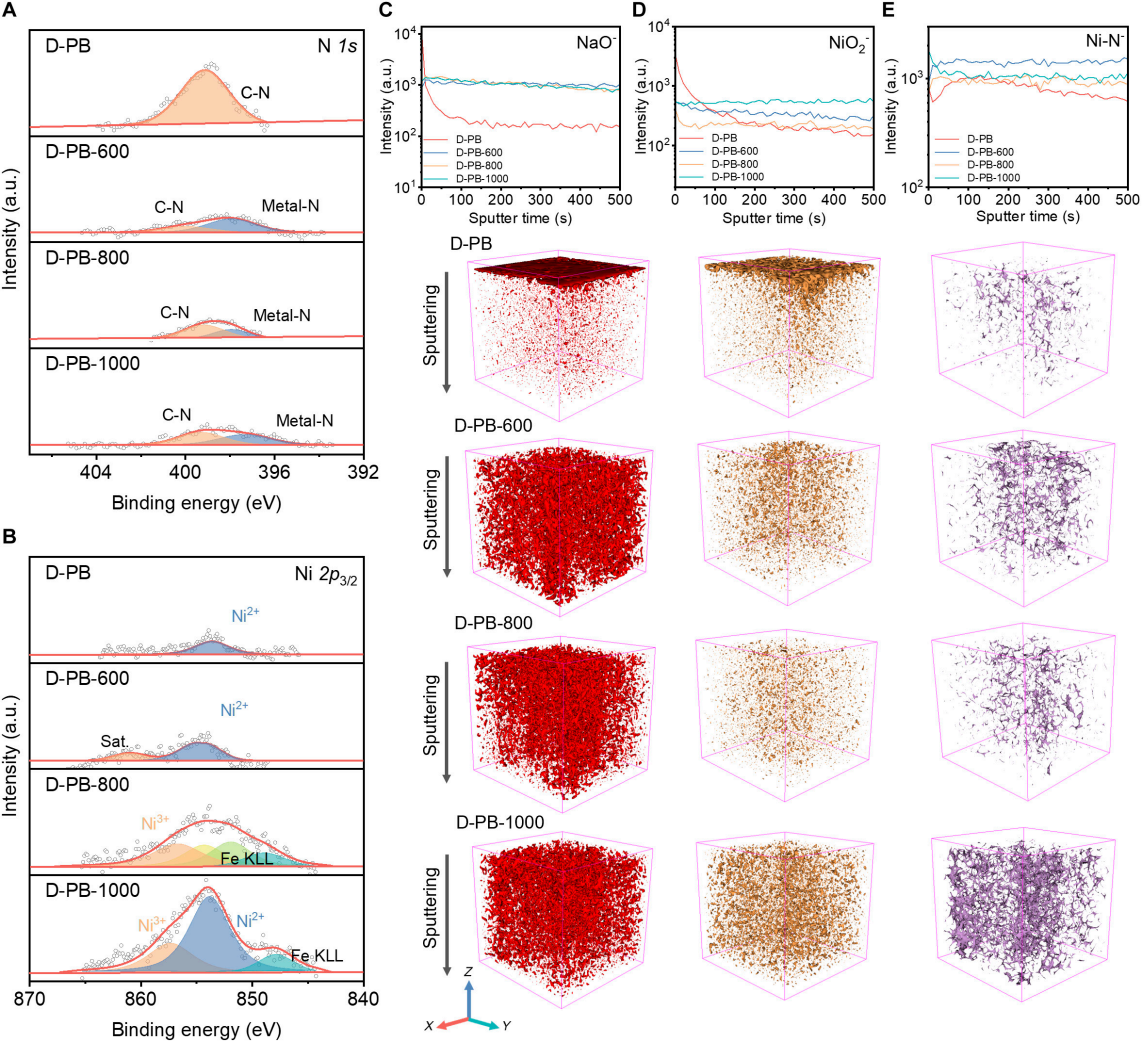
XPS and TOF-SIMS results of D-PB, D-PB-600, D-PB-800, and D-PB-1000. (A) N 1s XPS spectra. (B) Ni 2p_3/2_ XPS spectra. TOF-SIMS depth profiles and 3D views of (C) NaO^−^, (D) NiO_2_^−^, and (E) Ni–N^−^.

XPS and time-of-flight secondary ion mass spectrometry (TOF-SIMS) are applied to investigate the changes in elemental composition during the thermal treatment process. The XPS spectra of C and N show that C≡N in D-PB decomposes after thermal treatment, with a substantial decrease in intensity (Fig. 3A and Fig. S4) [26]. Notably, the C≡N in D-PB reacts with metals at the material interface at different temperatures, forming metal–N bonds. After thermal treatment, iron in D-PB oxidizes, while the manganese content shows negligible changes compared to the D-PB sample. The Fe 2p_3/2_ spectra of D-PB and D-PB-600 exhibit characteristic peaks at 706.84 and 709.54 eV (Fig. S5), indicating that iron in the PB materials exists as both elemental and +2 valent states. Following thermal conversion in air, the Fe 2p_3/2_ spectra show characteristic peaks at 709.23 and 711.35 eV, indicating that the oxidation states of iron transition to +2 and +3.

The Mn 2p_3/2_ spectrum shows a characteristic peak for +2 oxidation state manganese ions at 641.0 eV (Fig. S6). The oxidation state of manganese ions does not change at different thermal treatment temperatures, indicating that high-temperature conversion does not alter the oxidation state of manganese ions. In contrast, the Ni element shows significant differences with changes in thermal conversion temperatures. The XPS spectrum of Ni indicates the presence of divalent nickel at 853.82 eV in both D-PB and D-PB-600 (Fig. 3B) [27]. When the thermal treatment temperature is increased to 800 and 1,000 °C, the nickel intensity on the material surface increases markedly, and a characteristic peak for trivalent nickel at 857.36 eV appears. The nickel element in the material exists in a mixed valence state of +2 and +3, while in D-PB-1000, nickel mainly exists in the +2 valence state. This is a typical characteristic of the O3 nickel–iron–manganese (NFM) oxide material.

The TOF-SIMS results are in agreement with the EDS results and also detect metal–nitrogen species on the surface of thermally treated D-PB, similar to the XPS results (Fig. 3C to E and Fig. S7). In D-PB materials, the content of metal oxides is low, and there is a small amount of Ni–N species. After thermal conversion at 600 °C, D-PB decomposes, resulting in irregular distribution of metal oxides in the material, which aligns with the previously mentioned EDS experimental results. After thermal treatment at 800 °C, there are some uneven distribution phenomena laterally because at this temperature, a large amount of metal oxide forms. However, due to insufficient energy, it cannot fully diffuse into the bulk, leading to local aggregation. As the temperature increases to 1,000 °C, metal oxides are uniformly distributed both longitudinally and laterally, indicating that at this temperature, formed metal oxides from D-PB are recombined to form a uniform structure. Based on the Ni–N^−^ depth profile curves, it can be observed that with the increase in thermal conversion temperature, the content of Ni–N^−^ increases. The content of Ni–N^−^ at the surface of D-PB-1000 materials increases to around 10 times than that of D-PB (Fig. 3E).

### Sodium source supplementation

**Fig. 4.**
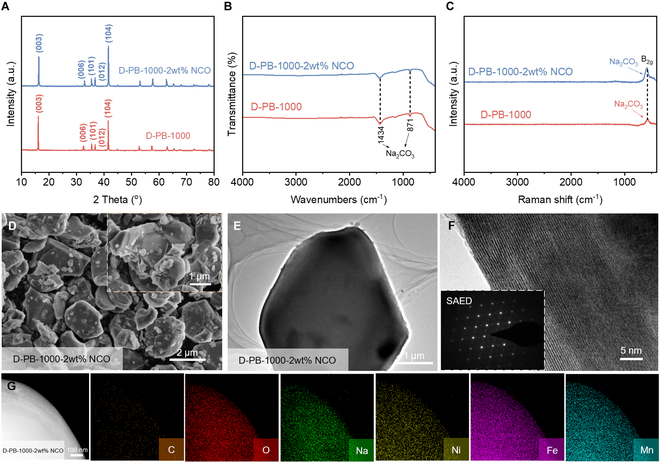
Characterizations of D-PB-1000 and D-PB-1000-2wt% NCO. (A) XRD spectra. (B) FTIR spectra. (C) Raman spectra. (D) SEM images of D-PB-1000-2wt% NCO. (E) TEM images of D-PB-1000-2wt% NCO. (F) HRTEM and SAED images of D-PB-1000-2wt% NCO. (G) EDS mapping of D-PB-1000-2wt% NCO.

During the thermal treatment, part sodium ions are lost, and a portion of the sodium cannot participate in the conversion of D-PB into metal oxides [28]. To address this, we supplement the sodium source 2 wt % Na_2_CO_3_ during the conversion of D-PB at 1,000 °C (D-PB-1000-2wt% NCO) to increase the sodium content in the target material. The structure and composition of D-PB-1000 and D-PB-1000-2wt% NCO are analyzed. The XRD results show that the addition of a sodium source does not change the crystal structure of the material after thermal conversion at 1,000 °C (Fig. 4A). Both the FTIR and Raman spectroscopy results for D-PB-1000-2wt% NCO are identical to those of D-PB-1000 (Fig. 4B and C), indicating that the sodium source supplement does not affect the structural composition of D-PB-1000.

Compared to D-PB-1000, D-PB-1000-2wt% NCO exhibits smaller particles, approximately 2 μm in size (Fig. 4D). The TEM images show a uniform and compact structure with intact edges (Fig. 4E). The HRTEM image reveals more clear and uniformly oriented lattice fringes, and the selected-area electron diffraction (SAED) image confirms that it belongs to a single-crystal structure, consistent with that of D-PB-1000 (Fig. 4F). The EDS image also confirms the uniform distribution of various elements within D-PB-1000-2wt% NCO (Fig. 4G). Therefore, it can be concluded that the sodium source supplement does not alter the uniformity of elements in D-PB-1000-2wt% NCO but changes the material's morphology and surface impurities.

### Electrochemical performance

**Fig. 5.**
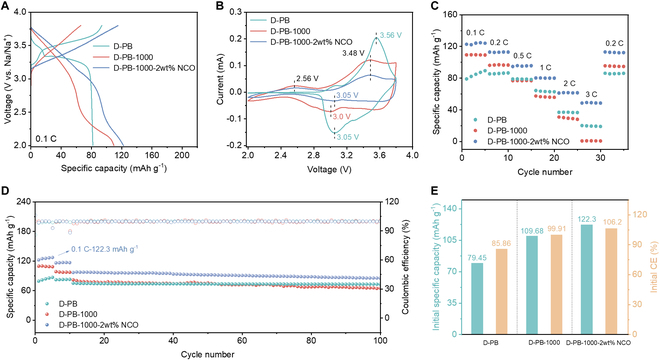
Electrochemical performances of D-PB, D-PB-1000, and D-PB-1000-2wt% NCO. (A) Voltage-specific capacity curves at 0.1 C. (B) Cyclic voltammetry curves. (C) Rate performances. (D) Cycling performances in the range of 2.0 to 3.8 V. (E) Summary of initial discharge specific capacity and Coulombic efficiency.

The materials formed at 1,000 °C exhibit a well-defined crystal structure, which is beneficial for the intercalation and deintercalation of sodium ions during charge and discharge processes [29]. To validate this hypothesis, we assemble sodium-ion half cells using these materials and evaluated their electrochemical performance. Figure 5A presents the initial charge–discharge curves at 0.1 C for half-cells containing D-PB, D-PB-1000, and D-PB-1000-2wt% NCO as cathode materials. The specific capacity of D-PB is only 79.45 mAh g^−1^. In contrast, the specific capacity of the thermal-treated D-PB-1000 material reaches 109.68 mAh g^−1^, and it further increases to 122.3 mAh g^−1^ for the D-PB-1000-2wt% NCO material, even surpassing the original PB material (98 mAh g^−1^). This demonstrates that thermal treatment can effectively enhance the electrochemical performance of the materials, and any sodium-ion losses incurred during the process can be compensated for by adding a sodium source. The supplementation of sodium sources leads to the formation of more complete layered structures. Hence, materials with sufficient sodium ions can provide high and stable discharge specific capacities. Additionally, the cyclic voltammetry (CV) curves of the D-PB-1000 and D-PB-1000-2wt% NCO materials show distinct oxidation–reduction peaks, indicating good cycle stability and reaction kinetics (Fig. 5B). Compared to D-PB, the D-PB-1000 and D-PB-1000-2wt% NCO materials exhibit lower potential differences between oxidation and reduction peaks, suggesting improved reversibility.

The materials subjected to thermal treatment at 1,000 °C outperform D-PB across the entire current density range from 0.1 to 3 C, particularly at high current densities (Fig. 5C). In contrast, D-PB materials treated at other temperatures exhibit negligible electrochemical activity. Among the upcycled materials, D-PB-1000-2wt% NCO shows the best performance, delivering a discharge specific capacity of 95.8 mAh g^−1^ at 0.5 C and maintaining a reversible capacity of 49.2 mAh g^−1^ even at 3 C, whereas D-PB-1000 loses capacity entirely at 3 C. The superior rate capability of D-PB-1000-2wt% NCO can be attributed to significantly reduced single-crystal particle sizes and Ni–N^−^ species on the surface. The cycle stability of the 3 materials at 0.5 C over 100 cycles is illustrated in Fig. 5D. Compared to D-PB-1000, D-PB-1000-2wt% NCO exhibits improved cycle stability, with a capacity retention rate of 86.1% after 100 cycles. The initial discharge specific capacities and Coulombic efficiencies of the materials are compared in Fig. 5E. D-PB-1000-2wt% NCO shows an initial discharge specific capacity of 122.3 mAh g^−1^ and a Coulombic efficiency of 106.2%, both surpassing those of D-PB (79.45 mAh g^−1^ and 85.86%). These electrochemical performances demonstrate that incorporating 2% Na_2_CO_3_ followed by thermal treatment at 1,000 °C in D-PB results in a structure that facilitates sodium-ion intercalation and deintercalation during charge–discharge processes, ultimately enhancing electrochemical performance.

### Environmental and economic analysis

**Fig. 6.**
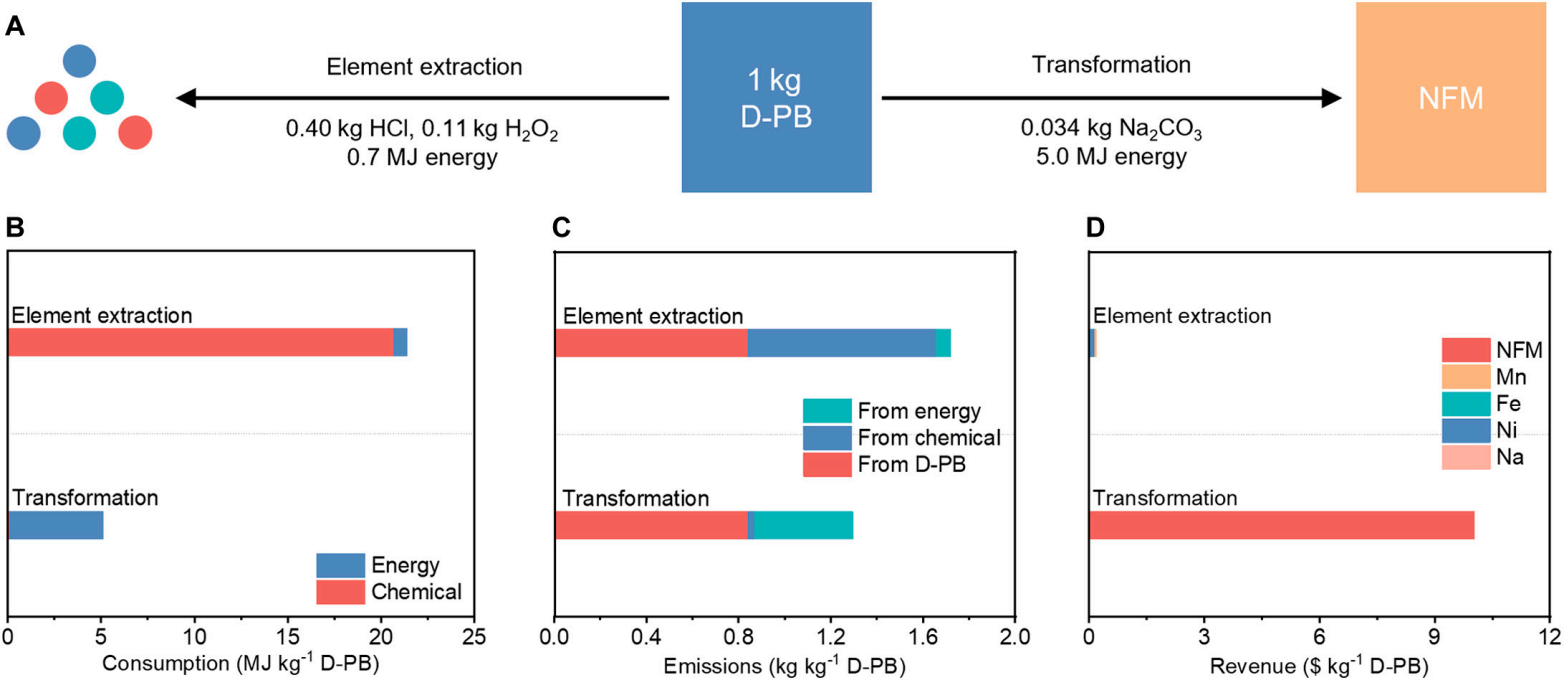
Environmental and economic analysis of element extraction and transformation. (A) Recycling routes of D-PB and corresponding consumptions. (B) Energy and chemical consumption. (C) Emissions from energy, chemical, and D-PB. (D) Revenue.

The feasibility of D-PB recycling depends on its environmental impact and economic benefits. Compared to element extraction, the D-PB transformation process demonstrates significant advantages in terms of chemical and energy consumption, emissions, and revenue. In element extraction, chemical consumption is assumed based on the minimum consumption in traditional metallurgical methods. For per kilogram of D-PB processed, about 0.40 kg of HCl, 0.11 kg of H_2_O_2_, and 0.7 MJ of energy are consumed (Fig. 6A and Table S1). When converting all chemical consumptions into MJ unit, element extraction requires 21.4 MJ kg^−1^ of D-PB, whereas transformation only consumes 5.1 MJ kg^−1^ of D-PB, with 5 MJ of that being the energy consumed during the thermal treatment (Fig. 6B). These consumptions correspond to 0.82 kg kg^−1^ D-PB for element extraction and 0.45 kg kg^−1^ D-PB for transformation in terms of gas emissions. Additionally, the release of 0.84 kg of emissions per kilogram of D-PB due to C≡N species increases the total emissions to 1.72 kg kg^−1^ D-PB for element extraction and 1.29 kg kg^−1^ D-PB for transformation (Fig. 6C). Importantly, the value of D-PB transformed into NFM is substantially higher than that of elements. The total value of metal elements in D-PB is 0.18 $ kg^−1^ D-PB, which is lower than the recycling cost of 0.26 $ kg^−1^ D-PB, resulting in a loss for recycling (Fig. 6D). In contrast, the transformation cost is only 0.13 $ kg^−1^ D-PB, while the recovered NFM is valued at 10.0 $ kg^−1^ D-PB. Therefore, the transformation method is economically viable.

## Conclusion

This study investigates the transformation of D-PB into layered cathode materials for SIBs through thermal treatment. D-PB undergoes thermal decomposition to form metal oxides, which are then recrystallized at higher temperatures to produce the layered structure. During the thermal process, additional elemental components can be incorporated into the D-PB to adjust the final composition of the layered material. For the final materials, there is a thin layer of metal–N substances on the surface, which enhances material's electrochemical performance. The environmental impact and economic analysis of the recycling process demonstrate that D-PB transformation has significant advantages over element extraction, particularly in terms of economic benefits. This makes it feasible to recycle low-value materials using the transformation strategy, potentially reducing the overall life cycle costs and environmental impact of SIBs in the future.

## Materials and Methods

### Materials

Battery-grade sodium metal foil, PB cathode material Na_2_Mn_0.6_Fe_0.2_Ni_0.2_[Fe(CN)_6_], anhydrous sodium hydroxide (NaOH, ≥98%), *N*-methylpyrrolidone (NMP, ≥99.5%), conductive carbon black (Super-P), polyvinylidene fluoride (PVDF), dimethyl carbonate (DMC; ≥99.9%), and glass fiber (Whatman GF/D) served as the separator are purchased from Guangdong Canrd New Energy Technology Co. Ltd. The electrolyte, 1.0 M NaClO_4_ in ethylene carbonate–diethyl carbonate (EC:DEC = 1:1 by volume) with fluoroethylene carbonate (5% by volume), is obtained from Suzhou Duo Duo Chemical Technology Co. Ltd.

### PB upcycling

PB materials exposed to air over 1 month become D-PB materials. The transformation of D-PB is conducted by thermal treatment of D-PB at different temperatures. Specifically, D-PB is heated in a muffle furnace to 600, 800, or 1,000 °C with a heating rate of 10 °C min^−1^ and maintained at this temperature for 10 h in the air atmosphere. Finally, it is allowed to cool naturally to room temperature. All the experiments are operated in the fume hood. The materials obtained are named D-PB-600, D-PB-800, and D-PB-1000 according to temperatures.

For the sodium source addition, D-PB is mixed with anhydrous NaOH in a molar ratio of 2%. The mixture is then heated in a muffle furnace at 1,000 °C for 10 h under the air atmosphere. The resulting material is named D-PB-1000-2wt% NCO.

### Battery assembly

The materials D-PB, D-PB-1000, or D-PB-1000-2wt% NCO are mixed with Super-P and PVDF in a mass ratio of 8:1:1. NMP is added as a solvent, and the mixture is stirred for 4 h to form a slurry, which is then uniformly coated onto aluminum foil. The prepared electrode is transferred to the 80 °C drying oven and dried for 12 h. The coin cells are assembled in a glovebox. The counter electrode uses sodium metal foil. A GF/D separator is used, and each coin cell contains approximately 70 μl of electrolyte.

### Electrochemical measurement

The electrochemical performance of D-PB, D-PB-1000, and D-PB-1000-2wt% NCO materials is evaluated using the following instruments and conditions. CV and electrochemical impedance spectroscopy (EIS) measurements are conducted using a VMP3 potentiostat (BioLogic). For CV tests, the scan rate is set to 0.1 mV s^−1^, and the potential range is from 2 to 3.8 V (versus Li/Li^+^). Battery tests are performed using a Land 2001A battery testing system, with the potential range set between 2 and 3.8 V.

The initial discharge/charge performance is tested at a rate of 0.1 C. Before long-cycle performance testing, the first 10 cycles serve as an activation process, alternating between 0.1 and 0.2 C. The long-cycle performance is then tested at a rate of 0.5 C. Rate performance tests are conducted at rates of 0.1, 0.2, 0.5, 1, 2, and 3 C.

### Characterization

The thermogravimetric analysis of the materials is performed on a thermal analyzer (STA449F3 NETZSCH, Germany) with a heating rate of 10 °C min^−1^. The crystal structures of the materials are analyzed using XRD on a D8 Advance Bruker instrument. The XRD measurements are conducted with Cu Kα radiation (λ = 0.154 nm) at 45 kV, 40 mA, and a scan rate of 10° min^−1^. FTIR is conducted using a Nicolet iS50 spectrometer (Thermo Scientific, Karlsruhe, Dieselstraβe, Germany) in attenuated total reflectance mode. Raman spectra are measured by a HORIBA LabRAM HR800 instrument. The surface elemental states of the materials are examined using XPS on a VersaProbeII ULVAC-PHI instrument. XPS analysis employs a monochromatic Al Kα x-ray source set at 15 kV, 25 W, and 100 μm, with depth profiling performed using 2-kV argon ion sputtering. TOF-SIMS analysis is carried out on a ULVAC-PHI nanoTOF-2 instrument, utilizing a Bi^3+^ ion gun at 30 kV and an Ar^+^ beam (3 keV, 100 nA) for sputter depth profiling at a rate of 0.1 nm s^−1^. The microstructural differences and crystallographic patterns of the materials are characterized using a field emission scanning electron microscope (FE-SEM, Hitachi SU-8010) equipped with an aberration-corrected high-angle annular dark-field detector (HAADF) and an HRTEM (FEI Tecnai G2 F30). The elemental content of the materials is determined using inductively coupled plasma optical emission spectroscopy (ICP-OES; Arcos II MV SPECTRO).

### Waste gas treatment

During the experiment, harmful gases such as hydrogen cyanide (HCN), nitrogen oxides (NO*_x_*), and carbon monoxide (CO) may be released. The fume hood system first collects the exhaust gas, which is then cooled and filtered to remove particles. All the gases undergo harmless treatment through selective catalytic combustion technology to reduce the harm to the environment.
