# Supplementary material for: Upcycling of Degraded Prussian Blue into Layered Materials for Sodium-Ion Battery
Source: Research (Wash D C). 2025 Mar 21;8:0643. doi: 10.34133/research.0643 (PMC11927955; doi:10.34133/research.0643)
Supplement: Supplementary 1 — Figs. S1 to S8 Table S1 [file research.0643.f1.pdf]

**Supplementary Materials**

# Upcycling of degraded Prussian blue into layered materials for sodium ion battery

Weng-Lam Wong[1,3], Jiahui Xu[1,3], Yun Zhao[1]*, Yadong Wang[1], Hao Du[1], Junhao Zhang[1], Yuqiong Kang[1], Yuqing Chen[2]*, Feiyu Kang[1], Baohua Li[1]*

**Affiliations:**

[1] Institute of Materials Research, Tsinghua Shenzhen International Graduate School, Tsinghua University, Shenzhen 518055, China

[2] Key Laboratory of Pollution Exposure and Health Intervention of Zhejiang Province, Interdisciplinary Research Academy, Zhejiang Shuren University, 310021, China

[3] These authors contributed equally.

*Correspondence to: yzhao.zjut@hotmail.com (Y.Z.); yuqingchen@zjsru.edu.cn (Y.C.); libh@mail.sz.tsinghua.edu.cn (B.L.)

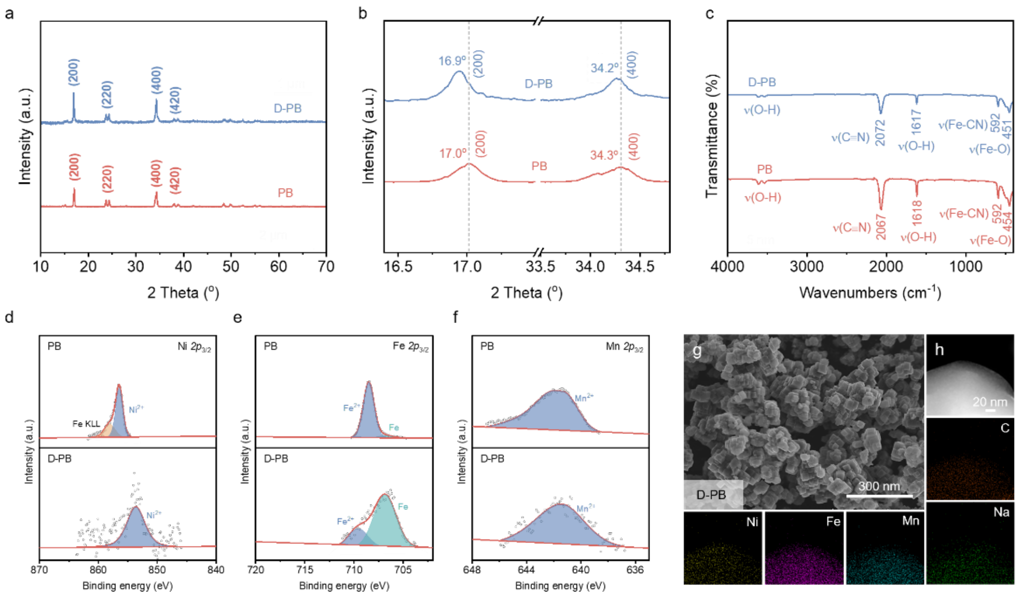


**Figure S1.** PB and D-PB characterizations. **a.** XRD spectra with 2 theta degree from 10° to 70°. **b.** XRD spectra with 2 theta degree from 16.4° to 34.8°. **c.** FTIR spectra. d-f. XPS spectra (**d.** Ni 2p 3/2. **e.** Fe 2p 3/2. **f.** Mn 2p 3/2). **g.** SEM image of D-PB. **h.** EDS mapping of D-PB.

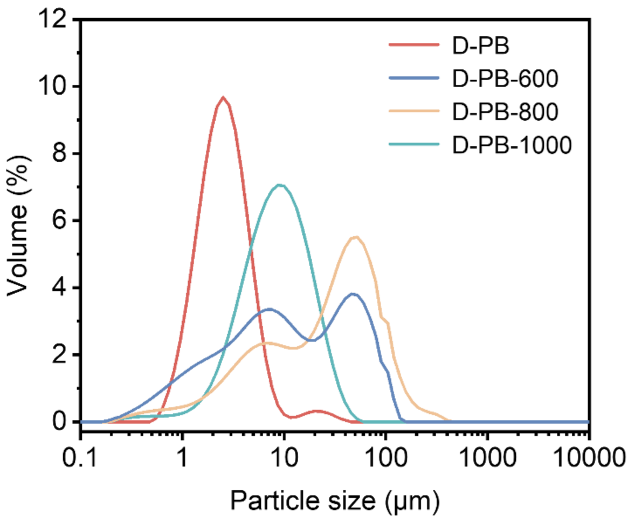


**Figure S2.** The particle size distribution of D-PB, D-PB-600, D-PB-800, and D-PB-1000.

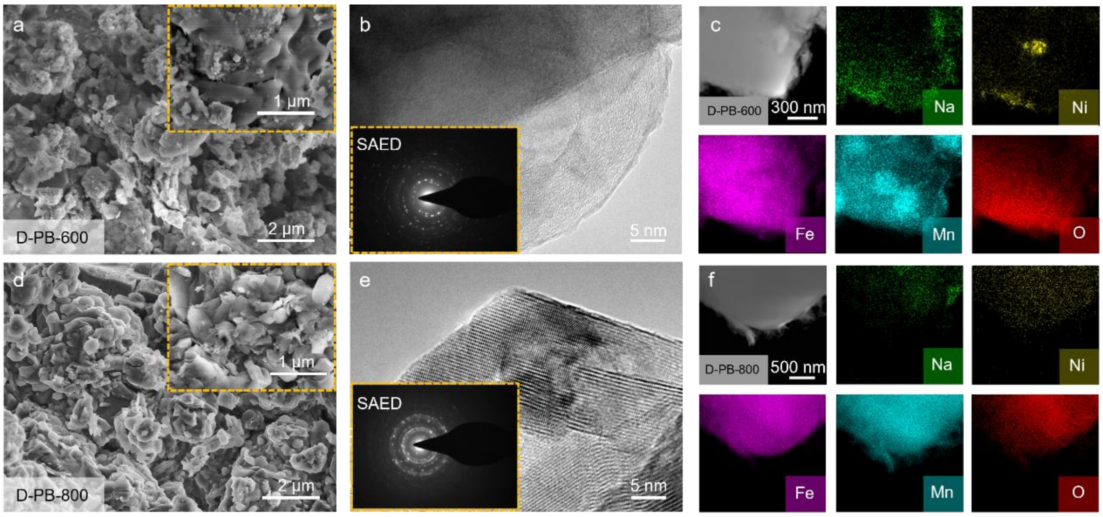


**Figure S3.** Morphology and micro-structure of the transformed materials. **a.** SEM images of D-PB-600. **b.** HRTEM and selected-area electron diffraction (SAED) images of D-PB-600. **c.** EDS mapping of D-PB-600. **d.** SEM images of D-PB-800. **e.** HRTEM and selected-area electron diffraction (SAED) images of D-PB-800. **f.** EDS mapping of D-PB-800.

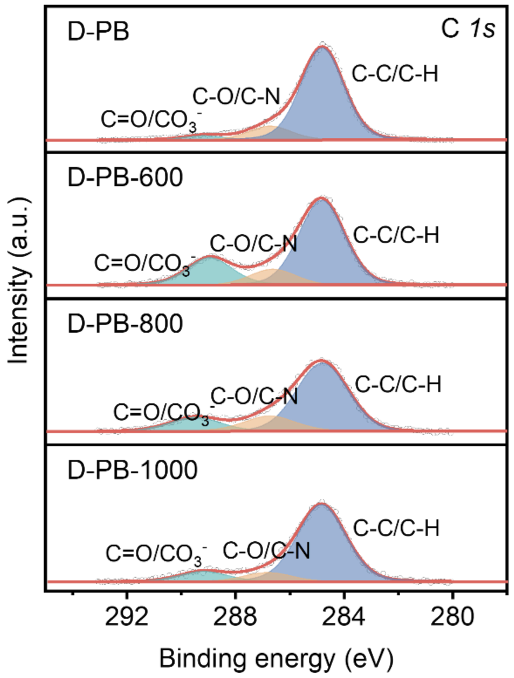


**Figure S4.** C 1s XPS spectra of D-PB, D-PB-600, D-PB-800, and D-PB-1000.

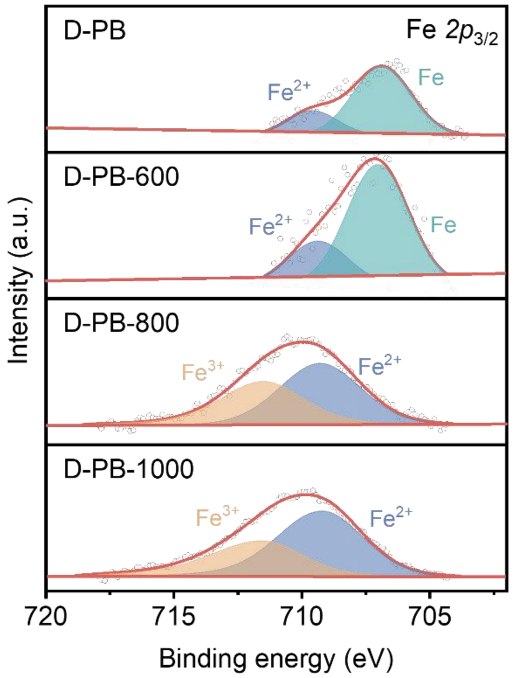


**Figure S5.** Fe $2p_{3/2}$ XPS spectra of D-PB, D-PB-600, D-PB-800, and D-PB-1000.

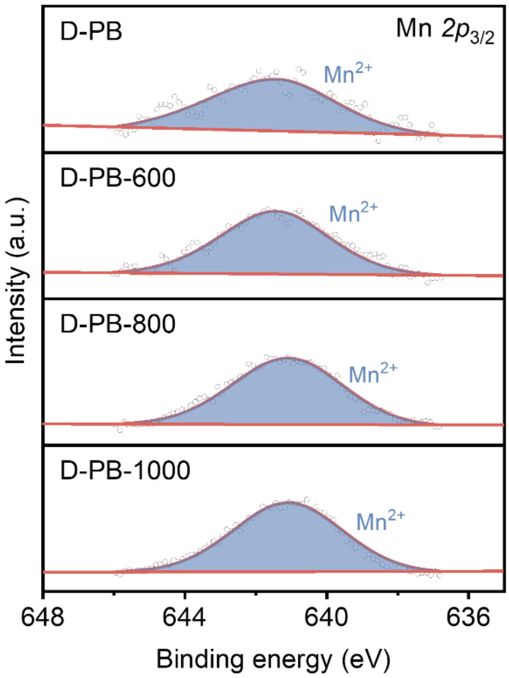


**Figure S6.** Mn $2p_{3/2}$ XPS spectra of D-PB, D-PB-600, D-PB-800, and D-PB-1000.

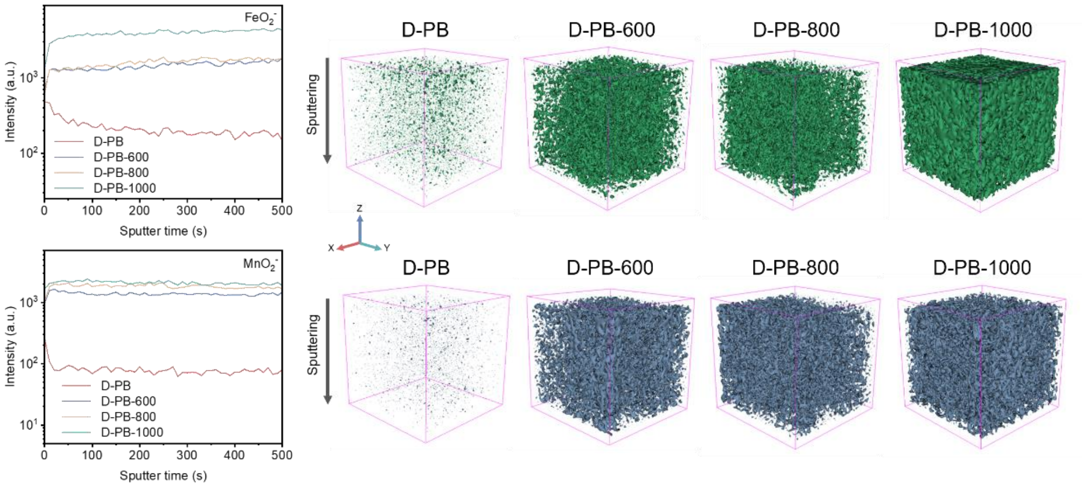


**Figure S7.** TOF-SIMs depth profiles and 3D views of $FeO_2^-$ and $MnO_2^-$.

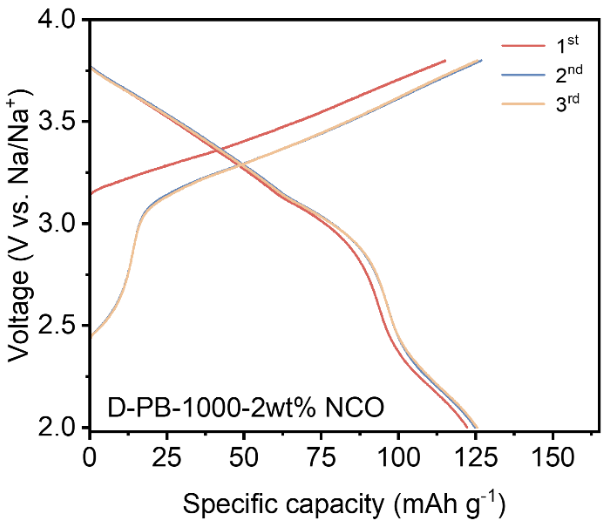


**Figure S8.** Voltage and specific capacity of D-PB-1000-2wt% NCO.

**Table S1.** Environmental and economic analysis of element extraction and transformation.

| **Element extraction** | | | | | | | |
|---|---|---|---|---|---|---|---|
| | Mass/kg | Revenue/$ | | | | | |
| Na= 14.66% | 0.1466 | 0.03372 | | | | | |
| Ni=3.74% | 0.0374 | 0.11508 | | | | | |
| Fe=21.36% | 0.2136 | 0.00986 | | | | | |
| Mn=10.51% | 0.1051 | 0.02425 | | | | | |
| | | 0.18291 in total | | | | | |
| | | | | | | | |
| | Materials/kg | | Carbon emission/kg | | Consumption/MJ | | Cost/$ |
| | HCl | 0.4 | from materials | 0.8415 | | | |
| | $H_2O_2$ | 0.11 | from chemical | 0.8176 | from chemical | 20.688 | |
| | | | from energy | 0.06 | from energy | 0.7 | |
| | | | | | | | 0.26 |
| | | | | | | | |
| **Transformation** | | | | | | | |
| | | Revenue/$ | | | | | |
| 1kg D-PB can obtain 0.70678kg | | 10.0363 | | | | | |
| | | | | | | | |
| | Materials/kg | | Carbon emission/kg | | Consumption/MJ | | Cost/$ |
| | $Na_2CO_3$ | 0.0338 | from materials | 0.8415 | | | |
| | | | from chemical | 0.027 | from chemical | 0.12168 | |
| | | | from energy | 0.426 | from energy | 5 | |
| | | | | | | | 0.13 |
| Price/$ $kg^{-1}$ | | | | | | | |
| Ni | Mn | Fe | Na | NFM | HCl | $H_2O_2$ | $Na_2CO_3$ |
| 3.1 | 0.23 | 0.046 | 0.23 | 14.2 | 0.43 | 0.69 | 0.15 |
